# The impact of effective communication-based care on the childbirth experience and satisfaction among primiparous women: an experimental study

**DOI:** 10.1186/s42506-022-00108-2

**Published:** 2022-08-09

**Authors:** Zahra Shamoradifar, Mohammad Asghari-Jafarabadi, Roghaiyeh Nourizadeh, Esmat Mehrabi, Hossein Namdar Areshtanab, Hoorieh Shaigan

**Affiliations:** 1 Student Research Committee, Tabriz University of Medical Sciences, Tabriz, Iran; 2 Department of Statistics and Epidemiology, School of Medicine, Zanjan University of Medical Sciences, Zanjan, Iran; 3 Center for the Development of Interdisciplinary Research in Islamic Sciences and Health Sciences, Tabriz University of Medical Sciences, Tabriz, Iran; 4 Department of Nursing and Midwifery, Faculty of Nursing and Midwifery, Tabriz University of Medical Sciences, Tabriz, Iran; 5 Department of Psychiatric Nursing, Faculty of Nursing and Midwifery, Tabriz University of Medical Sciences, Tabriz, Iran; 6 Department of Nursing and Midwifery, Guilan University of Medical Sciences, Rasht, Iran

**Keywords:** Birth experience, Birth satisfaction, Effective communication, Support and Control in Birth

## Abstract

**Background:**

There is insufficient scientific evidence on the effect of communication skills of childbirth care providers on maternal childbirth experience and satisfaction. The present study aimed to determine the effect of communication-based care on the childbirth experience and satisfaction among primiparous women.

**Methods:**

A total of 80 primiparous women participated in this experimental study who were randomly assigned into the intervention and control groups. According to the World Health Organization (WHO) care model, the intervention group received effective communication-based care, and the control group received the routine care. Data were collected using demographic and obstetric questionnaires, Labor Agentry Scale (LAS) and Birth Satisfaction Scale-Revised (BSS-R), and Support and Control in Birth (SCIB) scale applied 12 to 24 h after the intervention.

**Results:**

After controlling the effect of confounding variables, the mean scores of childbirth experience (51.23(1.54) and satisfaction (26.03(0.81) in the intervention group were significantly higher than that in the control group (45.33 (1.54) and 22.66 (0.81) respectively; [adjusted mean difference (AMD) = 5.90, *CI* = 95%: 1.17 to 10.62, *P* = 0.01] versus *AMD* =3.37, *CI*: 95%: 0.87 to 5.87, *P* = 0.001].

**Conclusion:**

Eeffective communication-based care improved childbirth experience and satisfaction of primiparous women. Therefore, it is recommended that health-care providers should be trained on the communication skills in the delivery room especially during a vital threatened crises such as the Covid pandemic.

## Introduction

Vaginal delivery is the most important event in women’s lives, which is accompanied by long-term emotional, social, and psychological effects lasting in their memory. Several predictors were known for childbirth negative experience and result in increased worries and anxieties in pregnant women [1, 2] and may induced an unpleasant childbirth experience. The unfavorable childbirth experience results in increasing the elective cesarean section to avoid possible negative experiences of vaginal birth [3]. Iranian women often prefer to give birth in a hospital, and according to the latest data from 150 countries, currently, 47.9% of all births occur by CS in Iran [2, 4].

In 2018, the WHO released the intrapartum care model to improve the quality of care for mothers and their babies during childbirth. The care model contains nine main components, including respectful labor and childbirth care, emotional support from a companion of choice, effective communication by staff, regular labor monitoring, documentation of events, and audit and feedback, pain relief strategies, oral fluid and food intake, mobility in labor and birth position of choice, pre-established referral plan, and continuity of care. One of the most important components is effective interaction with women and their families [5].

The maternity care staff’s level of communication skills was reported to be moderate in the early studies conducted in Iran [6, 7]. Unfortunately, the care model during labor and delivery is mainly physician centered in Iran. Although most of the WHO care model components are provided to pregnant women during delivery [5], the effective and acceptable interaction between midwives and pregnant women does not occur, due to the lack of one-by-one and midwife-led care. Furthermore, due to the midwives’ shift changes during the day and the lack of emphasis on interactive skills development for health-care providers, it is difficult to establish acceptable communication between the care providers and pregnant women. Therefore, it seems that identifying appropriate and effective intervention methods can help to improve the childbirth experience and satisfaction.

Very limited studies have been conducted to investigate the impact of communication-based care between the pregnant women and care providers on the maternal perceived support, experience, and satisfaction with birth process. In addition, the direct effect of communication skills of midwife and pregnant women on labor experience has not been addressed [8–10]. A systematic review (2018) reported that there is not sufficient evidence to support the impact of communication on maternal birth experiences, and the quality of the limited available evidence is very poor. Therefore, further studies should be conducted to evaluate the effect of midwives’ communication on the pregnant women’s birth experiences [11].

Considering the lack of studies about the effect of communication skills of care providers on the childbirth experience and satisfaction and according to the emphasis of the WHO on increasing the number of physiological deliveries by creating a pleasant childbirth experience and reducing avoidable maternal mortality, the present study aimed to determine the impact of effective communication-based care on the childbirth experience and satisfaction of primiparous women during the COVID-19 pandemic.

## Methods

### Study design

This posttest control group design was conducted on all women referring to the Shahid Nourani Hospital in Talesh, located in the north of Iran, for childbirth from September to December 2020. The inclusion criteria were primiparous women with singleton and a term pregnancy with gestational age of 37–42 weeks. The exclusion criteria included high-risk pregnancies (including placenta previa, placenta abruption, preeclampsia, and mother’s systemic diseases, non-cephalic presentation, having a history of any mental or physical illness, neonatal abnormality, and intrauterine fetal death).

### Sample size

The sample size was calculated according to the study of Hodnettt et al. (2013) [12] and based on the childbirth experience and satisfaction variables using G*Power software. Considering *m*_1_ = 39.0 (mean score of childbirth experience) and *m*_2_ = 46.8 with the assumption of 20% increase due to the intervention, *SD*_1_ = *SD*_2_ = 10.6, *α* = 0.05, and power = 80, the sample size was calculated 30 per group. According to the pilot study and regarding *m*_1 =_ 19.90 (mean score of childbirth satisfaction), *m*_2_ = 23.88 with the presumption of 20% increase due to the intervention, *SD*_1_ = *SD*_2_ = 4.14, *α* = 0.05, and power = 80%, a sample size of 18 was obtained per group. The sample size estimated based on outcomes of the study (childbirth experience and satisfaction and finally the higher sample size) was selected because we need the adequate sample size to enhance the study’s reliability. The final sample size was estimated to be 40 in each group considering 25% attrition.

### Sampling

Almost 150 pregnant women were screened from July to October 2020. Finally, 80 eligible women were assigned into the control and intervention groups using the random allocation software (RAS) through blocked randomization with a block size of 4 and 6 and a 1:1 allocation ratio. To conceal the allocation, the type of intervention was written on paper and placed in opaque envelopes numbered consecutively and prepared by a person not involved in sampling. A noninvolved person in the sampling process opened the envelopes.

### Data collection tool

#### The descriptive information form (DIF)

The demographic and obstetric questionnaire included the variables of age, educational level, occupation, duration of labor, history of abortion, type of delivery, and postpartum bleeding.

#### The Labor Agentry Scale (LAS)

The Labor Agentry Scale (LAS) questionnaire (Persian version) was used to assess the participants’ feelings and experiences during delivery. This 10-item scale, including six positive and four negative questions, is scored on a 7-point Likert scale ranging from 1 (never or rarely) to 7 (almost often). The sum of the scores ranges from 10 to 70. The higher the score, the more likely it is to have a positive birth experience. The Cronbach’s alpha coefficient is reported to be 0.98 [12]. In the Persian version of the questionnaire, the correlation coefficient of test-retest was reported to be 0.97, and the Cronbach’s alpha coefficient was obtained 0.94 [13].

#### The Birth Satisfaction Scale-Revised (BSS-R)

The Persian version of the Birth Satisfaction Scale-Revised (BSS-R) questionnaire with 10-item and three sub-scales was used to evaluate the women’s birth satisfaction. The first sub-scale includes 4 items was about the quality of care, the second sub-scale includes 2 items about the personal characteristics of the mother, and the third sub-scale includes 4 items about the stress of childbirth. The responses are rated on a 4-point Likert scale ranging from strongly agree (4) to strongly disagree (0). The total score of the instrument is between 0 and 40, and a higher score indicates greater satisfaction level. The Cronbach’s alpha coefficient of the questionnaire was 0.79 [14]. In the Persian version, the Cronbach’s alpha estimated for the whole tool was 0.74, and the alpha of the three sub-scales ranged from 0.698 to 0.801. ICC for determining reliability was 0.77 [15].

#### The Support and Control in Birth (SCIB) scale

The Support and Control in Birth (SCIB) questionnaire was used to measure women’s perception of birth support and control. The questionnaire contains 33 items, and its 3 sub-scales include internal control, external control, and professional support. Its internal control subscale includes 10 items of controlling pain, emotions, and behavior; the external control subscale includes 11 items of controlling pain about decisions and procedures. Furthermore, the professional support sub-scale consists of 12 items focusing on attitude, patience, empathy, and coping with pain. The questions are graded on a 5-point Likert scale ranging from strongly agree to strongly disagree. A score of 1 indicates less control and support, and a score of 5 represents more control and support. The items 1, 2, 6, 7, 10, 14, 17, 28, 29, and 32 are scored negatively. The range of the scores is between 33 and 155. The Cronbach’s alpha coefficient of the tool is reported to be between 0.86 and 0.93 [16].

In the present study, the reliability of the questionnaire of participants’ experiences, birth satisfaction and Support and Control during Birth was measured by determining internal consistency (Cronbach’s alpha coefficient), which was calculated at 0.90, 0.85, and 0.87, respectively, for the LAS, BSS-R, and SCIB questionnaires.

### Intervention

The researcher (first author) provided effective communication-based care for the intervention group (*n* = 40) during the active phase of labor (dilatation more than 4–6 cm of the cervix) and accompanied the patient throughout labor and delivery. The effective communication between caregiver and pregnant women was performed based on the WHO care model checklist [5]. The researcher introduced herself and provided information to the pregnant women and their companion concisely and understandably and avoided medical terms and used pictures and shapes when necessary. In order to communicate and introduce the delivery procedure, the researcher respected and responded to women’s needs, preferences, and questions with a positive attitude and supported their emotional needs with empathy through encouragement, praise, confidence, and active listening and provided shared decision-making for women. The written informed consent form was obtained from participants, for pelvic examination and other interventions. The researcher encouraged pregnant women to express their needs and preferences, kept women and their companion constantly informed of what was happening, answered their questions specially about the COVID- 19, and assured them about the confidentiality of all times. It is worth mentioning that the control group (*n* = 40) received routine care during labor and birth. The standard care includes a brief explanation of processes during labor; mostly, the midwife decides for an intervention (induction with oxytocin, pain relief, etc.).

### Statistical analysis

The SPSS version 24 software was used for data analysis at a significance level of 0.05. The Kolmogorov-Smirnov test assessed the normality of data distribution, and all data had a normal distribution. ANCOVA was used to evaluate the effect of the intervention, compare the mean scores of childbirth experience and satisfaction after the intervention, and control the effect of confounding variables (childbirth satisfaction was considered as confounder for childbirth experience and support and control; delivery type and induction with oxytocin were considered as confounder for childbirth satisfaction). The *t*-test was used to evaluate the effect of the intervention and compare the mean score of support and control in childbirth.

## Results

**Fig. 1:**
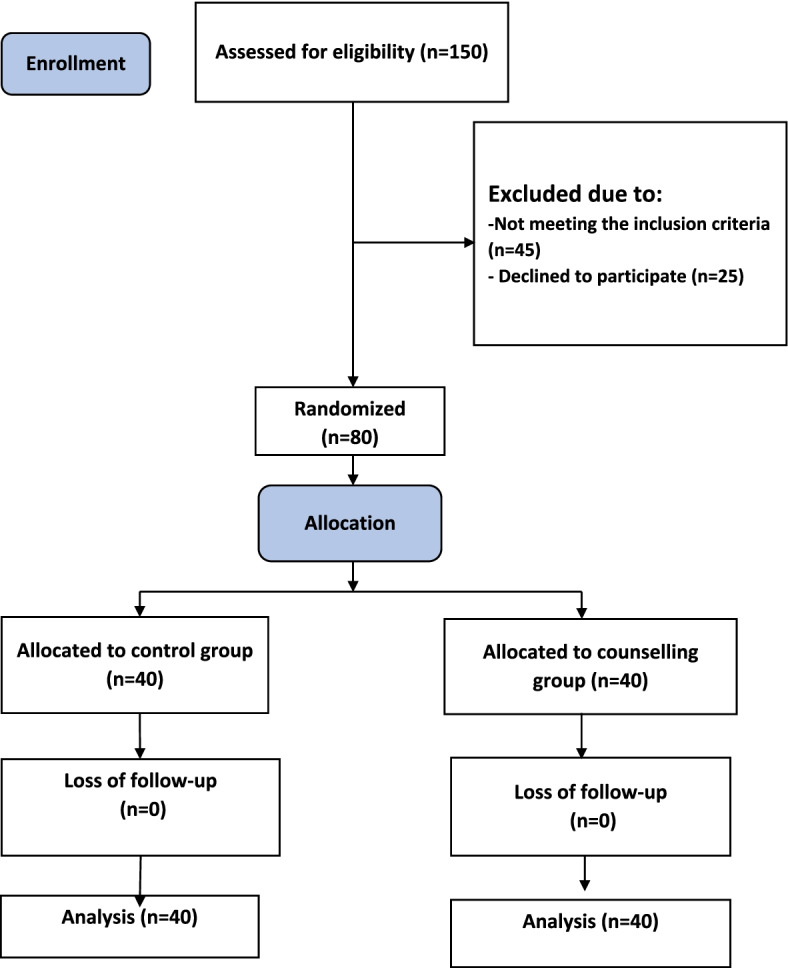
Flow chart of the study

The data obtained from 80 participants were statistically analyzed. Figure 1 indicates the flow chart of the study.

### The participants' demographic and obstetric characteristics

The mean (SD) age of participants was 25.40 (5.47) years in the intervention group and 24.68(5.82) in the control group, and most of the participants 93% (37) in the intervention group and 94% (38) in control group) were housewives. The mean (SD) of labor length of women was 7.96 (2.90) h in the intervention group and 6.48 (6.40) in the control group, and there was no statistically significant difference between the study groups in terms of labor length (*P* = 0.15). About two-thirds of women in the control group and nearly half of women in the intervention group had a physiologic delivery. Furthermore, two women (0.5%) in the control group and one (2.5%) in the intervention group had a cesarean section, due to arrested labor. In general, there was no significant difference between the two groups in terms of demographic and obstetric characteristics (Table 1).

**Table 1:** Demographic and obstetric charasteristic of parturient women (*n* = 80) in the studied groups (Talesh, Iran, 2020)

Variable	Intervention group (*n* = 40) *N* (%)	Control group (*n* = 40) *N* (%)	***p***-value
Age (year)	25.40 (5.47)	24.68 (5.82)	0.56*
Husband’s age	29.00 (5.29)	29.43 (6.67)	0.75*
Education			0.09**
Illiterate	4 (10.0)	6 (15.0)	
Less than diploma	13 (32.5)	19 (47.5)	
Diploma	12 (30.0)	13 (32.5)	
BSc and undergraduate	11 (27.5)	2 (5.0)	
Husband’s education			0.8**
Illiterate	4 (10.0)	4 (10.0)	
Under diploma	12 (30.0)	17 (42.5)	
Diploma	16 (40.0)	13 (32.5)	
**Employment status**	8 (20.0)	6 (15)	
Housewife	38 (94.0)	38 (95.0)	1.00**
Employed	3 (6.0)	2 (5.0)	
**Family income level**			
Enough	2 (5.0)	4 (10.0)	1.00**
Some extent	31 (77.5)	32 (80.0)	
Not enough	7 (17.5)	4 (10.0)	
**Accelerated delivery**			
Yes	5 (12.5)	10 (25.0)	0.12**
No	35 (87.5)	30 (75.0)	
**Shoulder dystocia**			
Yes	1 (2.5)	2 (5.0)	1.00**
No	39 (97.5)	38 (95.0)	
**Laceration grades 2 and 3**			
Yes	1 (2.5)	0 (0.0)	1.00**
No	39 (97.5)	40 (100)	
**Treatment of postpartum bleeding**			
Without intervention	40 (100)	38 (0.95)	0.49**
Misoprostol	0 (0.0)	2 (5.0)	1.00**
**Macrosomia**			
Yes	2 (5.0)	1 (2.5)	1.00**
No	38 (95.0)	39 (97.5)	
**Induction**			
Yes	18 (45.0)	15 (37.5)	0.15**
No	22 (55.0)	25 (62.5)	
**Wanted pregnancy**			
Yes	38 (95.0)	37 (92.5)	1.00ф
No	2 (5.0)	3 (7.5)	
**Is the sex of the fetus the mother’s favorite?**			
Yes	37 (92.5)	35 (87.5)	0.71**
No	3 (7.5)	5 (12.5)	
**Is the sex of the fetus the father’s favorite?**			
Yes	36 (90.0)	35 (87.5)	1.00**
No	4 (10.0)	5 (12.5)	
**Labor length**	7.96 (2.90)	6.48 (7.96)	0.15*
**Long labor**			
Yes	10 (25.0)	8 (20.0)	0.79**
No	30 (75.0)	32 (80.0)	
**History of abortion**			
No	38 (95.0)	35 (87.5)	1.00**
History of 1 abortion	2 (5.0)	5 (12.5)	
**Type of delivery**			
Physiologic	19 (47.5)	27 (67.5)	0.11**
Cesarean	1 (2.5)	2 (5.0)	
NVD with induction†	20 (50.0)	11 (27.5)	
**Postpartum hemorrhage**			
Yes	0 (0.0)	2 (5.0)	0.49**
No	40 (100)	38 (95.0)	
**Low APGAR**			
Yes	0 (0.0)	2 (5.0)	0.49**
No	40 (100)	38 (95.0)	
**Amniotomy**			
Yes	21 (52.5)	17 (42.5)	0.11**
No	19 (47.5)	23 (75.5)	
**Analgesia**			
Yes	8 (20.0)	4 (10, 0)	0.34**
**Other pain reliefs**			
No	32 (80.0)	36 (90.0)	
Yes	17 (42.5)	15(37.5)	

*Independent *T*-test .**Chi-square test. †Normal vaginal delivery. Variables were reported by number and percentage, except those reported by mean and standard deviation

### The perceived support and control during childbirth

After the intervention, the mean (SD) of perceived support and control during childbirth in the intervention group (123.5 (2.53)) was significantly higher than that in the control group 106.28 (2.57), [adjusted mean difference (AMD) = 17.21, *CI* 95%: 9.40 to 25.03, *P* = 0.001]. The mean (SD) of the internal control sub-scale was 29.9 (0.73) in the intervention group and 28.9 (0.74) in the control group after the intervention [*AMD* = 0.93, *CI* 95%: −3.07 to 1.18, *P* = 0.38], and this difference was not statistically significant. The mean (SD) of the external control sub-scale in the intervention group was 38.2 (1.08), which was significantly higher than that in the control group 31.2 (1.08) after the intervention [*AMD* = 7.04, *CI* 95%: 3.92 to 10.16, *P* < 0.001]. In addition, after the intervention, the mean (SD) of professional support sub-scale in the intervention group (42.99 (0.71)) was significantly higher than that in the control group (38.6 (0.71)) [*AMD* = 4.39, *CI* 95%: 2.32 to 6.45, *P* < 0.001] (Table 2).

**Table 2:** Comparison of support and control scores during delivery and its sub-domains in the intervention and control groups

Variable	Intervention group (Mean ± SD)	Control group (Mean ± SD)	AMD (*CI* 95%)^#^	*p*-value
Support and control in childbirth total (31–155)				
After	123.5 (2.53)	106.28 (2.57)	17.21 (9.40 to 25.03)	0.001*
Internal control subdomain (10–50)				
After	29.9 (0.74)	28.9 (0.73)	0.94 (−3.07 to 1.18)	0.38
External control subdomain (9–50)				
After	38.2 (1.08)	31.2 (1.08)	7.04 (−3.92 to −10.16)	0.03*
Professional support subdomain (9–50)				
After	42.99 (0.71)	38.6 (0.71)	4.39 (2.32 to 6.45)	0.001*

^#^ANCOVA test with control of baseline values. ^#^Mean difference with confidence interval of 95%. *Significant at *p*<0.05

### The childbirth experiences

After controlling the effect of support and control in childbirth, the mean (SD) of the scores of childbirth experience in the intervention group was significantly higher than that in the control group (51.23 (1.54) versus 45.33 (1.54), [adjusted mean difference (AMD) =5.90, *CI* 95%: 1.17 to 10.62, *P* = 0.01].

### The childbirth satisfaction

The mean (SD) of the scores of childbirth satisfaction in the intervention group was significantly higher than that in the control group after controlling the effect of support and control in birth (26.03 (0.81) versus 22.66 (0.81), [*AMD* = 3.37, *CI* = 95%: 0.87 to 5.87, *P* = 0.001] (Table 3).

**Table 3:** Comparison of childbirth experience and satisfaction score during delivery in the intervention and control group

Variable	Intervention group (Mean ± SD)	Control group (Mean ± SD)	AMD (CI 95%)^@^	*p*-value
Childbirth experience (acquired score 10 to 70)				
After intervention	51.23 (1.54)	45.33 (1.54)	5.90 (1.17 to 10.62)	0.001*
Childbirth satisfaction (acquired score0 to 40)				
After intervention	26.03 (0.81)	22.66 (0.81)	3.37 (0.87 to 5.87)	0.001*

*ANCOVA test with control of baseline values. ^@^adjusted mean difference (confidence interval of 95%)

## Discussion

The study’s findings indicated the positive effect of communication-based care on birth experience, satisfaction, and perceived support and control during childbirth. Providing effective communication-based care promotes a sense of control and support during childbirth, leading to a positive childbirth experience and satisfaction.

Based on the assessment of the research team, few interventional studies have been carried out on the impact of effective communication on the birth experience, satisfaction, and perceived control. The following studies highlight the importance of effective communication of care providers in the delivery room.

Chang et al. (2018) in their review study entitled “interventions to support the effective communication between maternity care staff and women in labour” reported that communication as an important component of care during childbirth helps to improve birth experiences, and they also mentioned that very low-quality evidence was found on effectiveness of communication training of maternity care staff [8]. Similarly, Chabbert et al. (2020) and Hauck et al. (2007) in a review study examined the predictors of the positive childbirth experience and reported that good communication of healthcare providers and midwives is an important predictor for childbirth experience [17, 18]. In another study, Dahlberg et al. (2013) investigated the impact of interpersonal communication on the childbirth experience and indicated that providing continuous care and paying attention to the quality of communication with pregnant women lead to a positive childbirth experience [19]. Furthermore, supporting pregnant women by delivery room staff and establishing a good relationship were revealed as an effective factor in the positive childbirth experience [3, 20–22]. These results are consistent with the results of the present study, which demonstrated the positive role of effective communication and support during the childbirth process. Therefore, it seems that more attention should be paid to the communication skills between care providers and pregnant women in the delivery room, which empower women for a pleasant vaginal birth.

Based on the results of the present study, communication-based care increases the birth satisfaction. Consistent with this finding, Dahlberg et al. (2016) found that the midwife’s behavior with pregnant women creates a sense of security in women and enhances their strength and belief in the management and control ability of themselves during childbirth [23]. Other similar studies examined the factors affecting childbirth satisfaction and reported that the communication between delivery care providers and pregnant women is one of the strongest predictors of childbirth satisfaction since the good communication is important in building trust among the hospital staff, especially midwives and pregnant women [24–29]. Furthermore, Baranowska et al. (2021) illustrated that midwives’ communication skills in creating childbirth satisfaction with labor and delivery are important. In addition, verbal and nonverbal communication is one of the effective issues in maternal satisfaction during childbirth [30]. The findings of other studies emphasized that communication-based training programs result in the empathy and sympathy between care providers and women during childbirth and assist in enhancing childbirth satisfaction [31–34].

The effective communication between caregivers and pregnant women during childbirth leads to a positive childbirth experience as a result of the ability to self-control during childbirth [23, 24], which is in line with the present study’s findings on the relationship between the childbirth experience and the sense of birth control in labor and delivery. Consistent with the present study results, other studies indicated a relationship between the feelings of control in labor and childbirth satisfaction. Additionally, using various aspects of care techniques in labor and delivery is important to meet the needs and expectations of pregnant women, create a sense of control during delivery, and increase delivery satisfaction [34–37].

Due to the lack of training on non-pharmacological pain relief methods during pregnancy for all mothers, the effective communication has no significant effect on the internal control in labor in the present study, which is mainly related to the ability of pregnant women to control pain and emotions during labor. This finding cannot be compared with other studies’ results, due to the limited studies conducted in this field.

### Limitations of the study

The present study results have limited generalization because it was conducted among a selected group of low- and middle-income women, in one hospital, and on women expected to deliver vaginally. Another limitation is related to the time of the intervention. The study outcomes were studied shortly after delivery, affecting delivery experiences. The effect of some forms of effective communication (such as training on non-pharmacological pain relief methods) could not be elicited because the intervention was conducted on women shortly before giving birth, while some forms of communication and support should be provided during pregnancy in order to influence the internal control in labor.

## Conclusions

Providing effective communication-based care improved the childbirth experience and increased the childbirth satisfaction and the sense of support and control by pregnant women during delivery. Although the provision of effective communication-based intervention generally influenced the perception of support and control during childbirth in the present study, it did not have a significant effect on the dimension of internal control. The results of present study can be used in the reviews to provide the best evidence-based research to achieve the appropriate care model during crisis. Regarding the importance of the findings of the present study, it is necessary to mention that Iran is one of the important countries with good level of health indicators in the Middle East region, and the findings of studies conducted in this region can be used as a model for countries with limited resources. It is recommended to improve all care providers’ skills of effective interaction with childbirth in the delivery room, provide care for pregnant women during childbirth based on all components of the care model introduced by WHO to improve the quality of care, and promote all aspects of support and control among pregnant women.

## Data Availability

Data are available on request due to privacy/ethical restrictions: The data that support the findings of this study are available on request from the corresponding author. The data are not publicly available due to privacy or ethical restrictions.

## Abbreviations

WHO: World Health Organization
DIF: The descriptive information form
LAS: The Labor Agentry Scale
SCIB: The Support and Control in Birth
MD: Mean difference
